# Encouraging bystander helping behaviour in a violent incident: a virtual reality study using reinforcement learning

**DOI:** 10.1038/s41598-022-07872-3

**Published:** 2022-03-09

**Authors:** Aitor Rovira, Mel Slater

**Affiliations:** 1 Department of Computer Science, University College London, London, UK; 2 Oxford Health NHS Foundation Trust, Oxford, UK; 3 Department of Psychiatry, University of Oxford, Oxford, UK; 4 Event Lab, Institute of Neurosciences, University of Barcelona, Barcelona, Spain

**Keywords:** Human behaviour, Computer science

## Abstract

Virtual reality (VR) affords the study of the behaviour of people in social situations that would be logistically difficult or ethically problematic in reality. The laboratory-controlled setup makes it straightforward to collect multi-modal data and compare the responses across different experimental conditions. However, the scenario is typically fixed and the resulting data are usually analysed only once the VR experience has ended. Here we describe a method that allows adaptation of the environment to the behaviours of participants and where data is collected and processed during the experience. The goal was to examine the extent to which helping behaviour of participants towards the victim of a violent aggression might be encouraged, with the use of reinforcement learning (RL). In the scenario, a virtual human character represented as a supporter of the Arsenal Football Club, was attacked by another with the aggression escalating over time. (In some countries football is referred to as ‘soccer’, but we will use ‘football’ throughout). Each participant, a bystander in the scene, might intervene to help the victim or do nothing. By varying the extent to which some actions of the virtual characters during the scenario were determined by the RL we were able to examine whether the RL resulted in a greater number of helping interventions. Forty five participants took part in the study divided into three groups: with no RL, a medium level of RL, or full operation of the RL. The results show that the greater extent to which the RL operated the greater the number of interventions. We suggest that this methodology could be an alternative to full multi-factorial experimental designs, and more importantly as a way to produce adaptive VR scenarios that encourage participants towards a particular line of action.

## Introduction

Under what circumstances will bystanders to a violent attack by one or more people on another intervene on the side of the victim to try to halt the violence? A dominant theory in this field is due to Latané and Darley^1,2^ who argued that group size, the number of bystanders to an incident, inhibits helping behaviour due to diffusion of responsibility. This has been verified many times, see the review by Fischer et al.^3^. Another approach focuses on the degree of social identity between the bystander and victim: the more that they share identity the greater the chance of helping intervention^4–6^. However, there are ethical problems in carrying out experimental studies that directly depict violence—it would be unacceptable to face unaware participants with a violent incident, for example, carried out by actors. Instead most research has focused on helping behaviour in non-violent emergencies. For example, it has been shown that football fans are more likely to help someone who appears injured in the street if they are wearing a shirt signifying that they support the same club as the fan, and to ignore those wearing the shirt of a rival club^7^. To further illustrate this point, the meta analysis by Fischer et al.^3^, included 105 studies with independent effect sizes. One of the criteria for inclusion was that the study depicted an emergency situation defined as “where a victim faced immediate danger, such as the victim injured him- or herself, or was threatened by a perpetrator” (p. 523). However, of the 64 emergency situations considered, the perpetrator was shown as present in the scenario in only 3 of them.

This situation of being unable to carry out controlled experimental studies that directly address the problem domain is similar to that of the famous experiments by Stanley Milgram on obedience to authority^8^. Notwithstanding the great importance of the topic, the conditions under which people will carry out harmful behaviour towards a stranger at the behest of authority figures, it is not possible to repeat Milgram’s studies given today’s ethics standards. Instead some authors have sought to mitigate some of the most extreme aspects of the Milgram study by only allowing electric shocks up to a relatively low (non-lethal) maximum^9^. Haslam et al.^10^ adopted a different approach whereby a group of professional actors and director were led to enact the electric shocks scenario. They were “not acting ‘as if’ they were an individual in the Milgram paradigm but rather ‘as’ a character who is then put in the Milgram paradigm.” In interviews subsequent to the performance the actors were queried about their thoughts and feelings during the scenario, which were similar to the reported findings amongst Milgram’s subjects.

An alternative method is to portray the scenario in immersive virtual reality (VR) where experimental participants are put into the role of what Milgram called the ‘Teacher’, and the ‘Learner’ is an entirely virtual human character. Here the Teacher is the one who calls out the questions and administers the shocks on wrong answers, and the Learner is the one answering and receiving the shocks. Slater et al.^11^ used such a VR setup, and found similar results to the original Milgram study, except with a lower level of stress exhibited by the participants. A further study^12^ showed that participants in such as setup often tried to help the Learner by giving them a clue about the correct answers, to avoid the unpleasantness of administering the shock and watching the painful response of the virtual Learner (who eventually demanded to be let out of the experiment).

At first sight it seems strange that people would respond to the pain of the virtual Learner, or even try to ‘help’ the Learner in VR since nothing real is happening—there is no Learner. However, a fundamental finding of decades of research into VR has shown that people do tend to respond realistically to virtual situations and events. This is related to the concept of ‘presence’ in VR, typically interpreted as the illusion of ‘being there’ in the environment depicted by the VR displays^13–16^. In^17^ it was argued that there are two orthogonal components, ‘Place Illusion’ (PI), the sense of ‘being there’ and a Plausibility Illusion (Psi), which is the illusion that the depicted events are really happening, even though the participant knows for sure that these are not true. PI is attributed to the mode of perception in VR being based on similar sensorimotor contingencies as perception in reality, where participants use their bodies to perceive (head turns, looking behind an object, bending down to look under, turning around to see or hear something behind, reaching out and touching)^18,19^. Psi is attributed to (i) the virtual environment responding to participant actions (for example, virtual characters move out of the way as the participant moves through a crowd), (ii) where the virtual environment initiates actions towards the participant (e.g., a character spontaneously looks towards or smiles at the participant), and (iii) the satisfaction of expectations of the participant when the VR depicts events that could happen in reality in which the participant is an expert. For example, Pan et al.^20^ found that medical doctors confronted by aggressive patients respond with stress, but also later they complained that they could not read the virtual computer on their desktop (since the resolution was not capable of displaying text at the size that would have been required for realism). This was a failure of expectation—(iii) above—but may also be related to PI since the resolution of the display was not high enough to fulfil a normal every day sensorimotor contingency (to see an object more clearly, move your viewpoint closer to it).

Experimental studies of bystander behaviour have also been carried out using virtual reality. The idea was proposed and illustrated with pilot data in^21^. In^22^ we examined the social identity hypothesis. Participants were all supporters of the Arsenal football team in the UK. In the VR they first had a conversation with a virtual human character (V) in a pub who either was an Arsenal supporter (in-group) (wearing an Arsenal shirt and speaking enthusiastically about the club) or a general football fan (out-group) (but not an Arsenal supporter). After a few minutes of this conversation another football fan (P) entered the pub, and verbally and ultimately physically attacked V for supporting Arsenal and wearing an Arsenal shirt. The response variable measured was the number of times that the participant intervened to help V. A second factor was whether V occasionally looked towards the participant as if for help. This occurred for one group of participants but not for another. Hence this was a 2 × 2 between groups design, with the factors being (in-group, out-group) and gaze (look-at, no look-at). It was found that the number of interventions was much higher for the in-group compared to the out-group, but that the gaze factor had no effect. However, if participants only *thought* that V was looking at them for help sometimes (even if this were not the case) then this increased the number of interventions.

In a further study^23^ we examined how the composition of a group of bystanders influenced the number of interventions. The setup was similar to one above, except that V was always in-group. There were three bystanders who were also all Arsenal supporters or not. It was found that the number of interventions was greater when the three bystanders were not Arsenal supporters. The explanation for this was diffusion of responsibility—if others were there who shared identity with the victim then it was just as much their duty to intervene as that of the participant. However, if all others present were not Arsenal supporters then the participant was the only one to have the duty to intervene to help the victim.

A further study using the same scenario, except with FC Barcelona fans rather than Arsenal, found that participants who were faster to want help a woman in distress (shown on a video) were later more likely to intervene in the football fan scenario between V and P^24^. This occurred under a condition where there was low cognitive load during the helping task, but not when there as high cognitive load.

Our previous work has mainly concentrated on the impact of social identity. Here we concentrate instead on how participants as bystanders respond to some specific actions of the protagonist (V and P) towards the participant and the actions of the virtual bystanders. We saw earlier that there is some evidence that if participants think that V is looking towards them for help that they are more likely to intervene. Here we follow that up with two further possible actions: the perpetrator P looking towards the participant and one of the three bystanders encouraging the participant to intervene. Instead of conducting a between group study we instead adopted a novel approach using Reinforcement Learning (RL)^25^, to answer the question as to whether a RL agent could learn to generate actions by V or P or the virtual bystanders that would encourage participants to intervene to help V. We also examined which of the three possible actions are more likely to result in participant responses.

Reinforcement learning has been used in VR (and more generally computer graphics) mainly as a way to create some aspect of the scenario, for example, Lopez et al.^26^ used deep RL for content creation, Conde et al.^27^ for animation of autonomous virtual agents, Elor and Kurniawan^28^ for agent movement guidance, and so on. Here we use RL to influence the behaviour of the human participants, to carry out tasks of which they were unaware. Kastanis and Slater^29^ showed that an RL agent could learn to influence people to move backwards along a straight line path to a particular place in the VR without them knowing that this was their goal. Effectively the agent learned about proxemics, that people will step backwards if another person (in this case a virtual one) approached them too closely. The agent even learned that if it was too far away from the participant for its forward movements to have an effect, it could wave to the participant to approach closer, and then its forward movements would induce the participant to step backwards. Rovira and Slater^30^ extended this to movements in a two dimensional space. Llobera et al.^31^ used RL to help participants choose a configuration of some factors in the VR that would maximise their preferences. The experiment described in this paper is a more complex social situation involving the actions of 5 virtual characters and the participant. The results nevertheless show that the RL agent can learn to get people to intervene, and also which actions might be best for this purpose.

## Material and methods

### Ethical approval

This study was approved by the UCL Research Ethics Committee, project ID 2707/001 and all methods were carried out in accordance with the corresponding guidelines and regulations in conformity with the Declaration of Helsinki. Participants had the scenario explained to them verbally, and also on a written information sheet. They were told that they could stop at any time without giving reasons, and gave written and informed consent.

### The virtual reality system

For this study, we used a Trimension ReaCTor, a projection-based VR system similar to the CAVE described by Cruz-Neira et al.^32^ We refer to this as the ‘Cave’. The use of a Cave is consistent with all the previous bystander studies that we have carried out^21,22,24,33,34^. The Cave used in this study consisted of a room with an approximate cubic shape with 3 walls and no ceiling, where the participant has freedom of movement. The rendered scene is displayed in stereo on all 3 walls and the floor with imagery connected seamlessly on the edges where two projection surfaces meet. The entrance is situated where the fourth wall should be. Walls are 3 m wide and 2.2 m high and are made of translucent material allowing the images to be projected from the other side. The floor is opaque, with the image projected from the top, with a size of 3 × 3 m. Projection surfaces receive the image from a projector through the mediation of a mirror. These mirrors are standard in Cave-like systems, they do not have a glass layer to avoid refraction and double reflections of the image. Additionally, mirrors make the system more compact by allowing the setup to fit in a smaller room.

The projectors used in our Cave were digital light processing projectors, model Christie DS + 6 K-M. Their native resolution is 1440 × 1050 pixels at a refresh rate of 100 Hz. The image projected on the floor needed to be cropped to 1100 × 1050 to adjust it to the surface dimensions. Pixel size is approximately 2.1 × 2.1 mm^2^ on the walls and 2.7 × 2.7 mm^2^ on the floor. The projectors were controlled by a PC cluster composed of four computers, each one equipped with an Nvidia Quadro FX 5600 graphics card, delivering stereoscopic graphics. The graphics cards were connected to a set of 6 infrared light emitters, strategically placed around the Cave to send the signal to the Crystal Eyes shutter glasses, also referred as goggles, that the user wore and provided them active stereoscopic vision. An Intersense IS-900 tracking system provided 6-degrees of freedom at a frequency rate of 120 Hz with accuracy between 2.0 and 3.0 mm. The IS-900 is a hybrid tracking system that utilises accelerometers and gyros to provide position and orientation data, with an ultrasonic ranging system for drift correction. The participant wore a small device attached to the goggles containing the inertial components as well as two small microphones to detect the ultrasonic chirps from a grid of emitters arranged above the volume of the Cave. The tracking system allowed for real time adjustments to the imagery according to the participant’s perspective, so he could look at the scene from different locations and angles.

### Experimental procedures

We recruited 45 participants through advertisements on the panel boards around the University and with an email we sent to all the students inviting them to participate in the study. It was advertised as a VR experience for Arsenal FC supporters and nothing was revealed at this stage about the nature of the scenario. People who were interested in participating completed an online form in which they answered questions related to football, such as specifying the Premier League (United Kingdom's top-level football league) team they supported and how often they watched the games on TV and attended the stadium. The recruitment criteria were based on their answers. We recruited male Arsenal F.C supporters of at least 18 years of age, who scored 4 or higher on the question “How much do you support your team?” on a Likert scale 1 (Not at all) to 7 (Very much so). The scenario was implemented only for Arsenal supporters, therefore any participant who supported a different Premier League team was not included. Female participants were not included to avoid gender variability and also because it was significantly harder to find candidates.

On the day of the experiment, participants arrived at a pre-booked time. They read the information sheet and signed the consent form. Once they were ready, they were guided to the Cave and the experience started. After the VR experience, they were asked to fill out a post-experience questionnaire and they were paid £7.

### Scenario

We used a version of the scenario similar to that described in the previous work above, involving five football supporters in a virtual pub. This was for two reasons. First, there is a strong association of violent behaviour and hooliganism in football, which can transcend socioeconomic boundaries and can occur domestically and internationally. Football fans may behave aggressively against people who support other teams or are from other nations. Second, it is straightforward to manipulate the affiliation of the virtual characters and simplifies the task of making the participant feel that he has a shared social identity with other characters by simply changing the appearance of the shirt they wear.

At the time booked for their session each participant individually entered the VR, where he found himself in a virtual bar on his own. Participants had 2 min to get used to wearing the goggles and acclimatise to the visual stimuli and the stereoscopic vision. During that time, the participant was instructed to look for objects related to football in the environment in order to acclimatise to the brightness of the virtual scenario and get used to the shutter glasses. After this, a first virtual character, the victim (V) entered the virtual bar through a door in front of the participant and walked towards the participant. This virtual human character wore an Arsenal football shirt. Once the participant acknowledged him, V started a conversation about the English Premier League team, Arsenal F.C. At some point during the conversation, a second man, the perpetrator (P), entered the scene and sat down by the bar, not far from where the participant and V were having their conversation. After exchanging their opinion about different matters related to Arsenal for about 2 min, P stood up and started to argue with V, accusing V of staring at him for no reason, while V responded that he was not looking at him. P asserted that he did not like Arsenal and people talking about them in the bar. V tried to defuse the situation by avoiding an escalation of the argument, but P’s behaviour became increasingly more aggressive. Finally, the situation became physical with P pushing V towards the wall, at which point the image faded out and the scenario ended. The confrontation ended after 2 min and 12 s from the moment P stood up.

**Figure 1:**
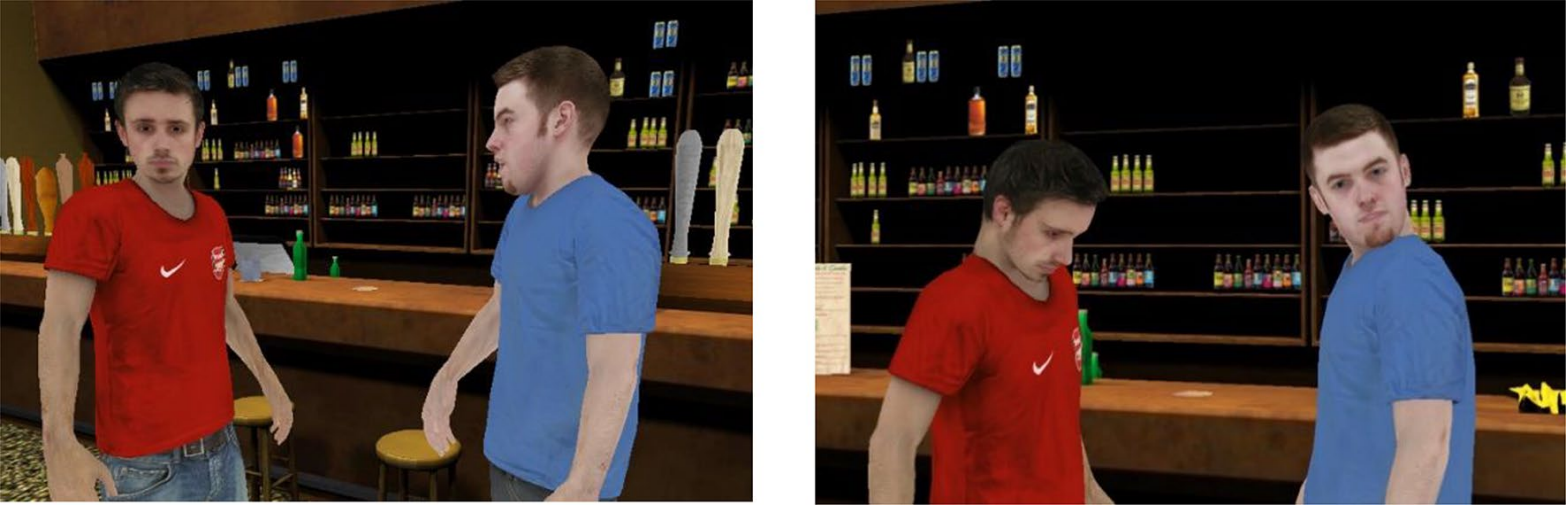
The character wearing the blue shirt verbally attacks the other one. Left—The victim (V) looks at the participant; Right—the perpetrator (P) looks at the participant.

There were three virtual bystanders in the scene. All three were initially sitting around tables and watching a TV sports program. Eventually, one of them stood up and got closer to the participant and the two people arguing. The argument is illustrated in Fig. 1. The video for the study described in^34^ shows the identical scenario but without the Reinforcement Learning^35^.

### Coding interventions

The response variables were determined by whether participants intervened to try to stop the argument and how many attempts they carried out. Any action, verbal or physical, was considered an intervention if it was carried out to get the attention of any the virtual characters in the scene. A verbal intervention was anything that the participant said to either V, P, or any of the bystanders in the virtual pub. Utterances that were not directed at these characters e.g., “think-aloud” utterances, did not count as an intervention. A physical intervention was considered as any attempt to make physical contact with any other characters in the scene, such as reaching out to them, moving close to them, walking in between victim and perpetrator to try to separate them, moving into their field of view to catch their eye, waving a hand, or any other hand gesture directed to them. If the participant stayed closer than 0.5 m to them, it was automatically considered a physical intervention.

A video camera provided a top-down perspective to observe how close the participant’s hands and body were to the virtual characters, and to correctly detect when he was turning his head to any of them. A researcher was watching the video stream and pressing the keys on a keyboard while there was a physical or a verbal intervention going on. The keys were pressed for as long as the intervention took place. This method was to notify the RL agent that an intervention was taking place, and the Q table was updated with the intervention counting just before the next action was chosen. This differs from our previous method of video coding we have detailed in previous studies^22,23^ where the coding was done offline, after the experience ended.

### Reinforcement learning

Reinforcement learning is a machine learning method where there is a goal to be achieved, an environment which can be in one of several states, and a set of actions that can change the state towards or away from or neutral with respect to the goal. At each discrete time step the RL agent determines an action which then may result in a change of state. Each action executed by the RL agent results in it receiving a reward that depends on whether the new state is further away from or closer to the underlying goal of the system (or unchanged). The reward is positive if the state moves closer to the goal, negative if it moves away, and possibly 0 or negative if there is no change. At first the RL agent chooses amongst possible actions at random. It builds up probabilities of how, for a given state, actions led to an intervention. Eventually it reaches a ‘policy’ which is a probability table associating actions in the context of states with subsequent changes in state, which are those most likely to lead towards the goal. This policy is derived to maximise the long term reward. In this study, that was equivalent to maximising the number of times participants intervened.

In our application in the context of the bystander scenario the state was determined by the distance between the participant and the two characters arguing. The distance was calculated from the participant’s head position and the middle point between the victim’s head and the perpetrator’s. The current state was determined when an action was about to take place. The distance was classified into three different states—Intervention, Active, and Passive. In the Intervention state, the participant was in the range of being able to reach out to V and P with his hands without having to first of all step towards them (distance < 0.5 m). In the Active state, the participant was close to them but keeping a short safety distance (0.5 m < distance < 1 m). In the Passive state, the participant stayed away from the confrontation (distance > 1.0 m).

There were three possible actions, each one performed by a different character: one by the victim, another by the perpetrator, and the last one by a bystander. The first action was performed 4 s after the confrontation started and the subsequent actions were carried out one every 10 s. The length of the argument, 2 min and 10 s, allowed for 13 actions in total for each participant.

The action VictimLookAt refers to V turning his head towards the participant, looking at him for 2 s before turning the head back to the original position to carry on with the scripted animation (Fig. 1). PerpLookAt was the same action but carried out by P. The last action, BystandersUtter, was that one of the virtual bystanders would say something out loud. All the utterances were designed to encourage the participant to intervene and they were performed sequentially. The list of utterances had 12 entries (Table 1) to ensure that the system would not run out of utterances even if all actions generated had been BystanderUtter. Some utterances were very similar but we used recordings from two different actors.

**Table 1:** Sequence of utterances that virtual bystanders might said out loud during the confrontation. Two different actors were used, so that where the expression is the same (e.g., 5 and 12) they sounded different.

1	“What is this guy doing?”
2	“Someone needs to do something about this!”
3	“This guy has lost it!”
4	“Tell him to calm down…”
5	“This guy is ridiculous!”
6	“Tell him to shut up!”
7	“Come on, who is going to tell him to stop?!”
8	“Someone needs to do something about this!”
9	“Who is going to tell him to stop?!”
10	“This guy has lost it…”
11	“Tell him to shut up!”
12	“This guy is ridiculous!”

We considered this as a ‘proof of concept’ study, to see whether it was possible that the RL would converge to a solution. Hence the number of actions and states are small. Additionally, the number of actions that could be tried for each participant was rather low, due to the short length of the confrontation. Reducing the time interval between actions would make the scenario look unrealistic, as an action could be repeated multiple times, and this could reduce plausibility and lead people to become detached from the scenario.

The outcome of each action was logged just before the next action was carried out. The reward was computed based on whether the participant had intervened during the 10 s window between actions. The reward was set to 1 if the participant intervened, either physically or verbally, making a maximum total of 2 if he intervened both physically and verbally. These were binary indicators, so that even if a participant intervened multiple times before the next action, the reward obtained for each type of intervention would still be 1 or 2. The reward returned was -0.1 in case an action did not lead to an intervention. This small negative reward was assigned to increase the chance of trying other actions that had not been tried before.

We used Watkins’ $Q(\lambda )$ as the RL algorithm^25,36^. This is a variation of the standard Q-learning algorithm that updates not only the $Q$ values of the state-action pair tried immediately before obtaining the reward, but also for previous pairs tried before that led to the reward. The $\lambda$ parameter controls how far back we go to update $Q$ values of state-action pairs tried in the past. Each state $s$ and action $a$ pair has one $Q$ value assigned to it*.* These values are updated every time an action led to a reward. In essence, the action with the highest $Q$ value in a given state is the action with the highest chance to lead to the highest reward. Therefore, that action is considered the current best action in that state.

The RL parameters were learning rate $\alpha =0.2$, discount factor $\gamma =1.0$ to make all interventions count equally independently of the moment they were carried out, and decay rate of the eligibility traces $\lambda =0.2$. Each experimental group had a predetermined epsilon $\epsilon$ value that specified the probability of each action to be chosen randomly. $\epsilon$ was predetermined before the VR session and remained constant throughout the experience. The results were accumulated over experimental groups but all participants within a group used the same initial $Q$ values and $\epsilon$.

The 45 participants were arbitrarily assigned to three groups with 15 in each. The first experimental group $(\epsilon =1)$ all started with all $Q$-values = 0. That means no prior experience was used, and all actions were chosen at random. Once all 15 participants from first group finished, their final $Q$-values were averaged and used as the initial $Q$-values for all the participants in the following group ($\epsilon =0.66$). And the same with the third experimental group ($\epsilon =0.33$) so the knowledge was accumulated over the groups.

### Implementation

The scenario was adapted from Rovira et al.^23^. In our previous study, once the argument started, the characters’ animations were scripted and there was nothing a participant could do to change the course of events. In this study, although the animations were still mainly scripted, we added the extra RL-determined layer so that the characters would perform the actions that were directed to the participant. This provided the illusion that they played a central role in the scenario aimed to enhance the feeling that they could do something about it.

### Experimental variables

There is one independent variable, *proprandom*, which is the proportion of randomness in the operation of the RL algorithm. Specifically, $\epsilon$ is the probability that each action will be chosen randomly and *proprandom* is the actual proportion of actions that were chosen randomly. In statistical terms, $\epsilon$ is the true mean of *proprandom*. If the number of samples were high enough, these two values would be equal. The variable *proprandom* varied between 0 and 1, with mean ± SD 0.33 ± 0.477, median 0.69 and interquartile range 0.54. When the level of randomness was 1 the RL did not operate at all and each action was selected at random, as described earlier.

The values of $\epsilon$ were designed to fall into three groups of equal size (n = 15 participants in each) according to how much RL learning was allowed: None (all actions were chosen uniformly randomly), Medium (where a proportion of the actions were chosen by some level of RL learning and the rest at random) and High, where the RL chose most of the actions through learning. This is shown in Table 2 with respect to *proprandom*. We refer to this factor as *RLLevel* (level of RL).

**Table 2:** The Distribution of *proprandom* by *RLLevel*, n = 15 in each group.

*RLLevel*	*Proprandom*			
	Mean	SD	Min	Max
None	1	0	1	1
Medium	0.72	0.138	0.46	0.92
High	0.33	0.130	0.08	0.46

The response variable (*resp*) is the number of helping intervention responses of any type to the 13 actions caused by the RL, and hence ranges between 0 and 13.

Operationally, our hypothesis is that this response variable should be negatively associated with *proprandom*. If this were the case then it would indicate that the more that the RL agent functions the greater the number of interventions, indicating the RL agent was able to induce interventions. Alternatively, we can consider the independent variable as the factor *RLLevel*, where we would expect greater values of *resp* for None, Medium and High in that order.

### Statistical models

Each action by the RL agent could result in a response or not by the participant. Since there were $N$ = 13 actions, the responses of each participant might be modelled as a binomial random variable, the number of responses out of 13. However, it is unlikely that the probability of a response would be constant across the 13 trials, since the participant may learn over time, and also the situation depicted became more aggressive. A distribution that can account for this variability is the Beta Binomial distribution, where the probability $\left(p\right)$ of responding is itself a random variable drawn from a Beta distribution, and then whether a response occurs is dependent on this probability. In this case the number of responses has a binomial distribution conditional on $p$. Hence the likelihood function for this model is:

1 $$\begin{aligned} resp_{i} & \sim BetaBinomial\left( {N,\alpha_{i} ,\beta_{i} } \right)\,\,\, \\ & \alpha_{i} ,\beta_{i} > 0\,\,\,\, \\ & i = 1,2, \ldots , n = 45 \\ \end{aligned}$$

Here ${\alpha }_{i},{\beta }_{i}$ are parameters with unknown values. The mean (expected value) of this distribution is:

2 $$E\left( {resp_{i} {|}N, \alpha_{i} ,\beta_{i} } \right) = \frac{{N\alpha_{i} }}{{\alpha_{i} + \beta_{i} }}$$

Let ${\alpha }_{i}={p}_{i}\phi$ and ${\beta }_{i}={(1-p}_{i})\phi$, for $\phi >0$ (the scaling parameter) then from (Eq. 2):

$$E\left({resp}_{i}|N, {p}_{i},\phi \right)=N{p}_{i}$$

Here ${p}_{i}$ is the probability of a response, which can be related to the independent variable *proprandom*, or to the factor levels of *RLLevel* (None, Medium, High).

For *proprandom* we have a linear logistic model:

3 $${p}_{i}= {inv\_logit(\beta }_{0}+{\beta }_{1}{proprandom}_{i})$$

where

$$inv\_logit(x)=\frac{1}{1+\mathrm{exp}(-x)}$$

The inverse logit function is necessary to ensure that the ${p}_{i}$ are in the range [0, 1]. This is a standard logistic model.

Similarly, for *RLLevel* we have:

4 $${p}_{i}= {inv\_logit(\gamma }_{0}+{\gamma }_{1}{M}_{i}+{\gamma }_{2}{H}_{i})$$

where

$$M_{i} = \left\{ {\begin{array}{*{20}l} {1,} \hfill & {if\, RLLevel = Medium} \hfill \\ {0,} \hfill & { otherwise.} \hfill \\ \end{array} } \right.$$

and

$$H_{i} = \left\{ {\begin{array}{*{20}l} {1,} \hfill & {if\, RLLevel = High} \hfill \\ {0,} \hfill & {otherwise.} \hfill \\ \end{array} } \right.$$

Note that ${\gamma }_{0}$ represents the influence of $RLLevel=None$, since this occurs when ${M}_{i}={H}_{i}=0.$

Solving these models with Bayesian methods is straightforward. We use weakly informative priors for the coefficients and the scale parameter^37,38^. These are priors that are proper probability distributions but with wide effective ranges. The priors are shown in Table 3 together with their 95% credible intervals.

**Table 3:** Prior distributions for the parameters.

Parameters	Prior distribution	Prior 95% credible interval (equal tails)
${\beta }_{j}, {\gamma }_{k}$	$Normal (mean=0, SD=10)$	− 20 to 20
$\phi$	$Gamma(shape=2, rate=0.1)$	2.4 to 55.7

The model was implemented using the Stan probabilistic programming language^39,40^ (https://mc-stan.org/) via the Rstudio interface (https://mc-stan.org/users/interfaces/rstan). The Stan implementation was run with 2000 iterations and 4 chains. The ‘leave-one-out’ (loo) library was used for model checking and cross validation^41^ (https://mc-stan.org/users/interfaces/loo). Stata 16.1 (https://www.stata.com/) was used for some figures and tables.

## Results

### The effect of the RL

**Figure 2:**
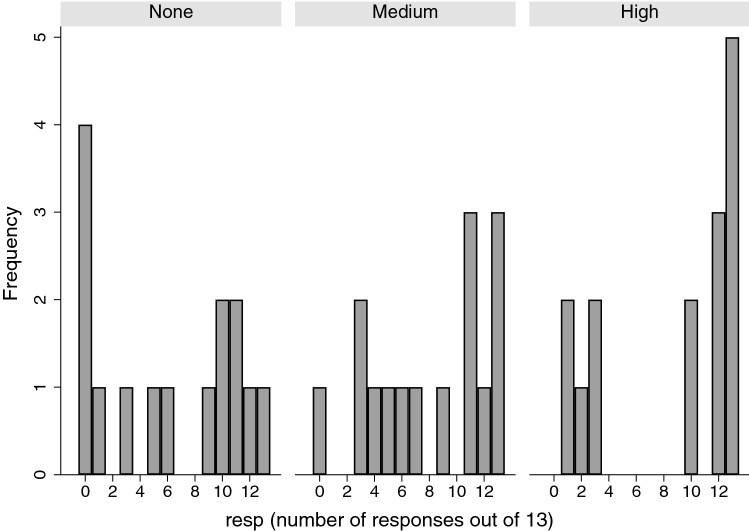
Histograms of *resp* by *RLLevel.*

Figure 2 shows the histograms of *resp*, the number of responses out of 13 by the *RLLevel* factor. The mode is 13 for the High level, 0 for None, and for the Medium level there is a tendency to be skewed towards a higher number of responses but not as great as the High level.

Table 4 shows the summaries of the posterior distributions for the two models (Eqs. 3, 4). First we consider the model with *proprandom* as the independent variable. The parameter ${\beta }_{0}$ is the intercept corresponding to *proprandom* = 0. The probability that this parameter is positive is 0.993. Moreover, ${\beta }_{1}$ is the slope of the linear predictor between *resp* and *proprandom*, and there is a high probability that this is negative, 1 – 0.023 = 0.977. Hence greater values of *proprandom* are associated with a smaller number of responses, or putting this positively, the more that the RL operated the greater the probability of interventions.

**Table 4:** Summaries of the posterior distributions of the parameters, showing their mean, standard deviations, and 95% credible intervals.

Parameter	Coefficient of	Mean	SD	2.5 percentile	97.5 percentile	Prob > 0
*Proprandom*						
${\beta }_{0}$		1.34	0.58	0.26	2.55	0.993
${\beta }_{1}$	*proprandom*	– 1.45	0.76	3.04	– 0.01	0.023
$\phi$		1.17	0.27	0.73	1.76	
*RLLevel*						
${\gamma }_{0}$		– 0.31	0.38	– 1.04	0.45	0.205
${\gamma }_{1}$	Medium	0.79	0.52	– 0.21	1.80	0.934
${\gamma }_{2}$	High	1.16	0.54	0.12	2.23	0.984
$\phi$		1.20	0.28	0.73	1.85	
						**Prob > 0.5**
${p}_{none}$		0.42	0.09	0.26	0.60	0.199
${p}_{medium}$		0.61	0.08	0.45	0.76	0.902
${p}_{high}$		0.70	0.08	0.54	0.83	0.988

Prob > 0 is the posterior probability of the parameter being positive. The last 3 rows refer to Eq. (5).

Now we consider the model of (Eq. 4) for *RLLevel* as the independent factor. In this case the coefficients for the Medium and High operation of the RL are well into the positive range, whereas the result for the random condition (None) has posterior probability of being negative of 1 – 0.205 = 0.795, although the 95% credible interval includes 0 well within its range. This further demonstrates that the more that the RL operated the greater the number of interventions.

**Figure 3:**
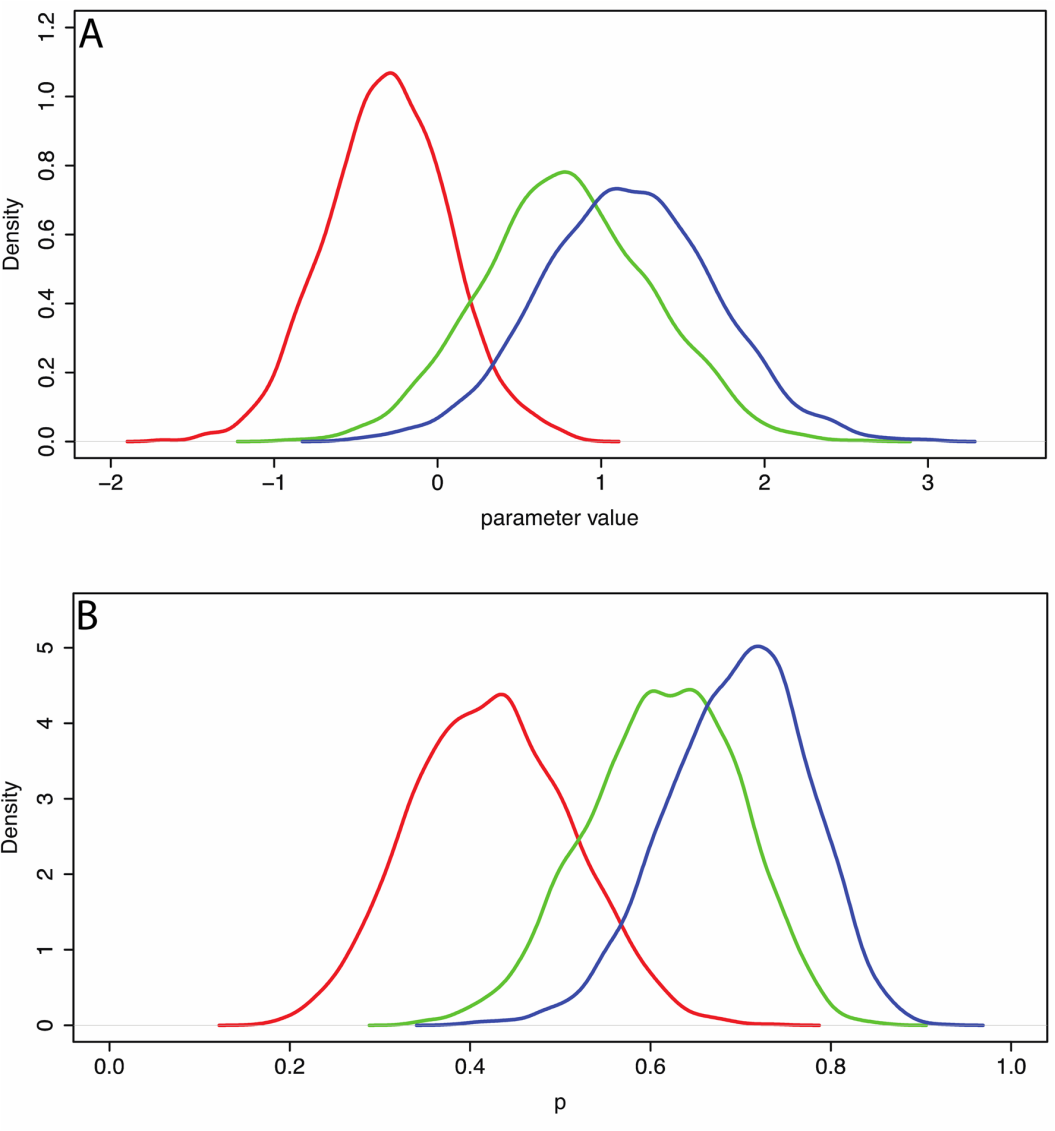
Posterior distributions of the parameters and probabilities. (**A**) The posterior distributions for ${\gamma }_{0}$ (red), ${\gamma }_{1}$ (green) and ${\gamma }_{2}$ (blue). (**B**) The posterior distributions of ${p}_{none}$ (red), ${p}_{medium}$(green), and ${p}_{high}$(blue) defined in Eq. (5).

Figure 3A shows the posterior distributions for the ${\gamma }_{j}$ showing a clear separation between the *RLLevel* None condition and the other two. For example, the posterior probability that ${\gamma }_{0}>1.5$ is 0, ${\gamma }_{1}>1.5$ is 0.096, and ${\gamma }_{2}>1.5$ is 0.262.

We can also compute the posterior distributions of the probabilities of a response under the *RLLevel* None, Medium and High conditions. Let

5 $$\begin{aligned} & p_{none} = inv\_{{logit\left( {\gamma_{0} } \right)}} \\ & p_{medium} = inv\_{{logit\left( {\gamma_{0} + \gamma_{1} } \right)}} \\ & p_{high} = inv\_logit\left( {\gamma_{0} + \gamma_{2} } \right) \\ \end{aligned}$$

Figure 3B shows the posterior distributions of these parameters, and the posterior probabilities that they are greater than 0.5 are shown in Table 4. As a further example, from these distributions, the posterior probability that ${p}_{none}>0.7$ is 0.001, ${p}_{medium}>0.7$ is 0.157, and ${p}_{high}>0.7$ is 0.505.

It should be noted that the posterior credible intervals are much narrower and focussed than the prior credible intervals, indicating that the data overwhelmed the priors.

The Stan simulation of these models converged without problem, with all Rhat = 1 indicating that the 4 chains were comparable and mixed. The ‘leave-one-out’ method^41^, a method that encapsulates repeated fits to the data with one observation left out each time, and all the others used to predict it, similarly indicated no problem with convergence or outliers.

**Figure 4:**
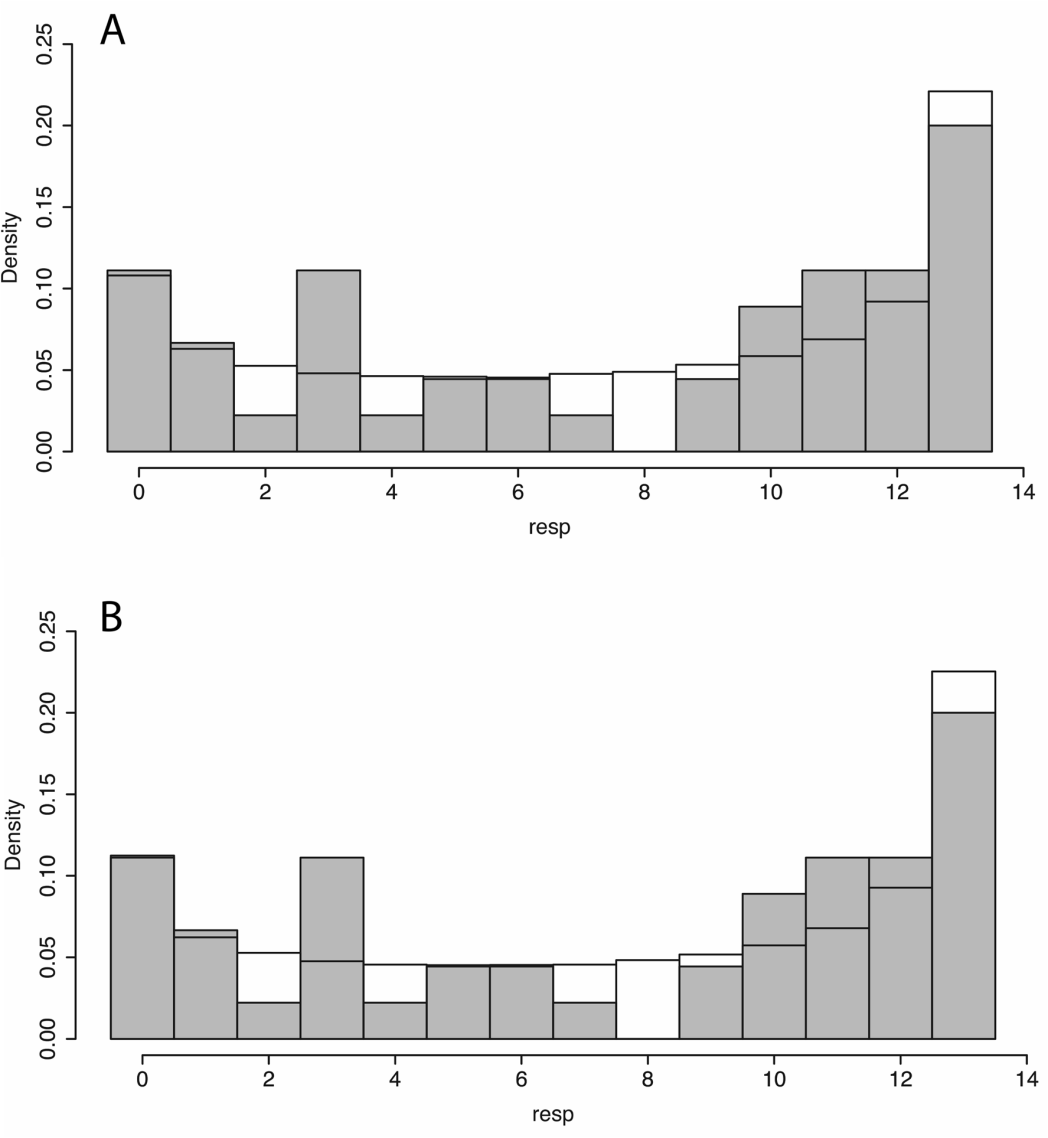
Histogram of the observed values of resp (in grey) overlaid with the histograms from the predicted posterior distributions (shown in white). (**A**) For the model with *proprandom* as the independent variable (Eq. 3). (**B**) For the model with *RLLevel* as the independent factor (Eq. 4).

The Stan programming language supports the simulation of new data from the posterior models. 4000 new observations were generated on each of the two models (Eqs. 3, 4). Figure 4 shows the histogram of observed values of *resp* overlaid with the histograms of the predicted posterior distributions. In each case the pattern of the predicted posterior matches the observed. If instead of the Beta Binomial distribution just the Binomial distribution were used, then the fit is a very poor one.

### The effects of the different actions

The analysis above shows that the more that the RL is allowed to operate beyond random, the greater the number of interventions. However, we can also examine how the different actions (VictimLookAt, PerpLookAt, BystandersUtter) impacted the likelihood of a response. For each of the 45 individuals we have 13 observations where there was an action followed by a response. Here we consider the response as binary—either they responded or did not.

Let ${y}_{i}=1$ if there was a response to an action and ${y}_{i}=0$ otherwise. Here $i=\mathrm{1,2},\dots ,585$ since there are 45 individuals each with 13 trials. Let ${vl}_{i}, {pl}_{i}, {bu}_{i}, i=\mathrm{1,2},\dots ,585$ each be 1 or 0 depending on whether the action was VictimLookAt, PerpLookAt, BystandersUtter, respectively. Note that for any $i$ only one of these three can be 1 and the rest will be 0.

Let ${p}_{i}$ = $P\left({y}_{i}=1\right)$ and hence ${1-p}_{i}$ = $P\left({y}_{i}=0\right)$. This is the Bernoulli distribution, denoted $bernoulli({p}_{i})$. The goal is to model variations in ${p}_{i}$ as a function of the type of action. We define the linear predictor:

6 $${\eta }_{i}={u}_{{id}_{i}}+{\beta }_{1}{vl}_{i}+{\beta }_{2}{pl}_{i}+ {\beta }_{3}{bu}_{i}$$

with the constraint

7 $$\sum_{j=1}^{3}{\beta }_{j}=1, { \beta }_{j}\ge 0$$

The constraint is necessary because the three independent variables are linearly related since ${vl}_{i}+{pl}_{i}+ {bu}_{i}=1$, all $i$.

${id}_{i}, i=\mathrm{1,2},\dots ,585$ is a vector denoting the identifier of the corresponding participant. In particular the vector $id$ ranges between 1 and 45 in blocks of 13, i.e., $id =$ [1,1,…,1, 2,2,…,2,…, 45,45,…,45].

${u}_{{id}_{i}}$ is a parameter that allows for possible individual differences between the participants (since the data includes 13 trials on each). Then,

8 $$\begin{aligned} & p_{i} = \frac{1}{{1 + e^{{ - \eta_{i} }} }} \\ & y_{i} \sim bernoulli\left( {p_{i} } \right) \\ \end{aligned}$$

which is a standard Bernoulli logistic model.

The prior for $({\beta }_{1},{\beta }_{2},{\beta }_{3})$ was chosen uniform over the simplex defined by (Eq. 7) (a Dirichlet distribution with all parameters 1). Each ${u}_{k }\sim normal(mean=0,SD=1)$, $k=\mathrm{1,2}, \dots , 45$.

Stan was used as above, except that 8000 iterations were needed (since there are many more parameters). The model converged with all Rhat = 1.

**Figure 5:**
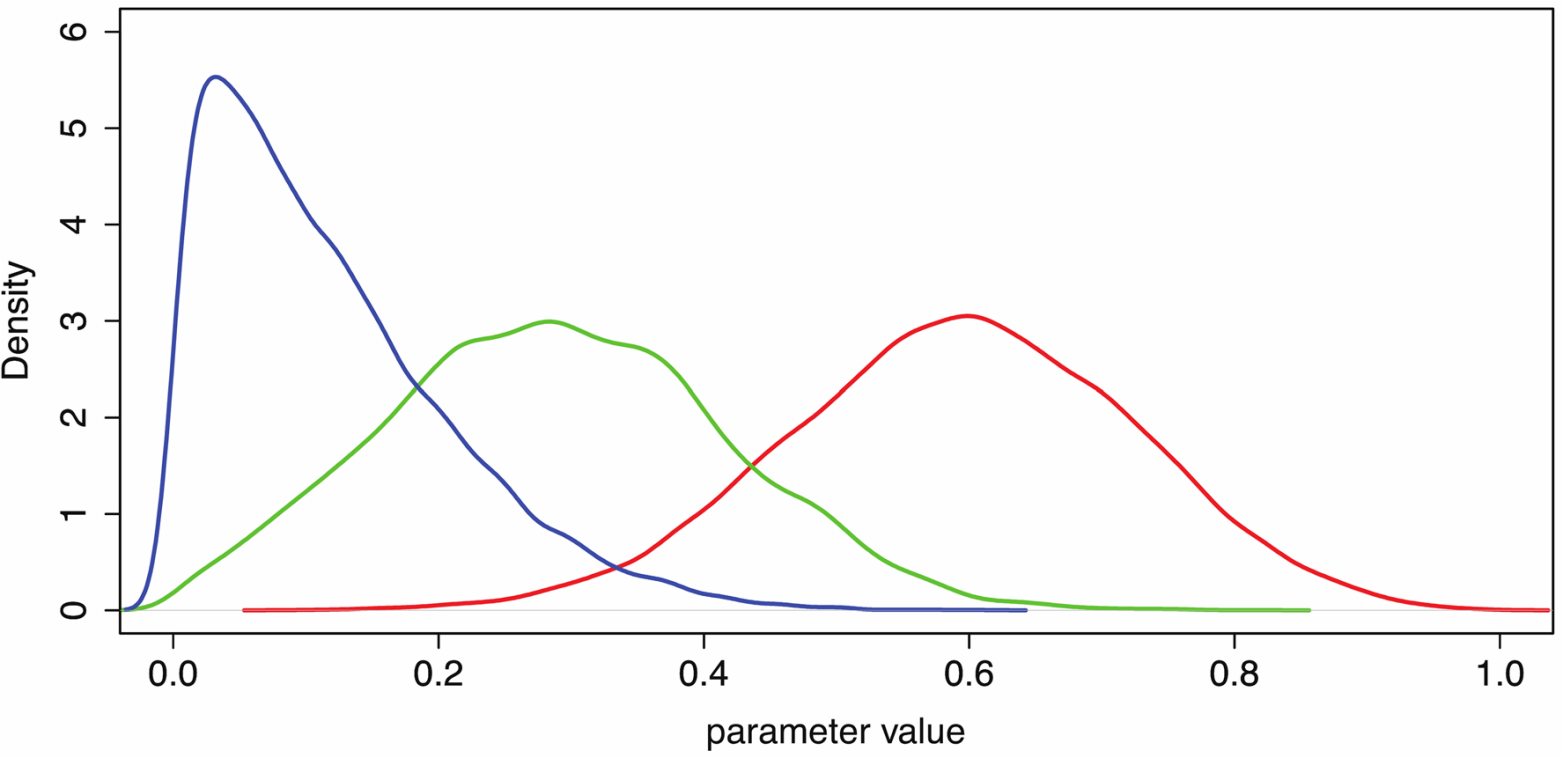
Posterior distributions of the parameters of model (Eqs. 6–8). Red: ${\beta }_{1}$ (VictimLookAt), Green: ${\beta }_{2}$ (PerpLookAt), Blue: ${\beta }_{3}$ (BystandersUtter).

Table 5 shows the summaries of the posterior distributions of the parameters. This shows that VictimLookAt has the greatest probability of leading to a response. Figure 5 shows the posterior distributions, where it clear that VictimLookAt has the greatest impact.

**Table 5:** Summaries of the posterior distributions of the parameters, showing their mean, standard deviations, and 95% credible intervals.

Parameter	Coefficient of	Mean	se_mean	2.5%	97.5%	*P*rob > 0.5
${\beta }_{1}$	VictimLookAt	0.59	0.13	0.33	0.84	0.763
${\beta }_{2}$	PerpLookAt	0.29	0.13	0.05	0.54	0.053
${\beta }_{3}$	BystandersUtter	0.12	0.09	0.00	0.34	0.001

Prob > 0.5 is the posterior probability of the parameter being > 0.5.

We generated 16,000 simulated observations on the response variable ${y}_{i}$ using the model, as in the previous section. The Point Biserial Correlation coefficient between the simulated and observed data is 0.75, with 95% confidence interval 0.72–0.79, indicating that the model fits the data well. The leave-one-out (loo) analysis yielded no problems of influential or outlying points.

## Discussion

We set out to investigate whether, during the course of an attack by a perpetrator on a victim in VR, participants could be encouraged in real-time to intervene to intervene. We adopted RL in order to examine whether the agent can learn over time which actions to generate to maximise the probability that the participant would intervene. If this succeeded a second goal was to see whether different types of action by the victim, perpetrator or virtual bystanders might have a differential effect on the likelihood of intervention by the participant.

On the first point we found that the more that the RL operated (i.e., the less random its determination of actions) the greater the number of interventions. This was shown in two ways: (i) by treating the actual proportion of random choices of actions used by the agent as the independent variable, and (ii) classifying the extent to which the RL operated into None, Medium and High. In both cases it was found that there is a negative association between the amount of randomness and the number of interventions. This analysis was carried out by analysing the numbers of responses out of 13 trials for each respondent. With respect to the second goal we found a markedly greater probability of an intervention response to the action of the victim looking towards the participant compared to the perpetrator looking and a bystander encouraging intervention.

In a recent meta-analysis of bystander studies that have used VR for experimental purposes or as a training tool only 11 studies (out of a possible 12,972) were found that satisfied this criteria^42^. Nine of these were experimental studies to examine factors that influence bystander behaviour, and 2 were trials of intervention programs which concentrated on presenting existing programs to encourage bystander intervention comparing the effect of using VR with video. Studies of factors that influence bystander behaviour have concentrated, as we have seen, mainly on external factors such as the size of the crowd, and also on social identity issues, for example^5^.

There is very little recent research on how the actions of the victim may impact bystander behaviour. Wilson^43^ carried out a study where a woman (confederate) standing in the street dropped a stack of computer cards. As male participants walked by, she randomly chose whether to explicitly ask for their help in picking them up and sorting them, or did not directly ask for help. The results showed that the participants were far more likely to stop to help if asked to do so. The results were explained as an example of how ambiguity of the situation can influence behaviour. It was argued that often bystanders do not know what to do, or whether their help is really needed or wanted, and might feel embarrassed to intervene inappropriately. However, when ambiguity is reduced (because the victim actually indicates the need for help) then they are more likely to intervene.

The importance of ambiguity in the likelihood of bystander interventions was originally studied by^44,45^ as a challenge to the view of Darley and Latané^2^ that the size of the crowd is the dominant factor. In several studies they found a critical role for ambiguity, the less ambiguous the situation (i.e., that someone needed help) the more likely that there would be an intervention, independently of crowd size. The ambiguity was in the nature of the emergency, whether it was clear or not that an emergency situation had really occurred. These studies did not actually depict a violent situation, but one where someone is in trouble and needs help (such as having fallen down and was apparently hurt). It emphasises the point of the utility of VR in presenting situations of aggression and near violence in which the participant is directly involved (both spatially, being in the same virtual space as the action, and through prior conversation with the victim).

In our study the emergency situation was clear and unambiguous, no one could have mistaken the argument between P and V as a friendly discussion between fans of different football clubs. However, whether the participant should intervene was open to question. At least 2 of the three actions would have reduced ambiguity about the need of V for help: V looking towards the participant, and another bystander encouraging action. P looking towards the participant is ambiguous—since it may be interpreted as a warning for the participant not to intervene. The action of V sometimes looking towards the participant, a direct indication from the victim of being aware of the presence of the participant, was more likely to lead to an intervention.

The work from nearly 50 years ago of proposing the level of ambiguity as an important factor contributing to the probability of bystander intervention has not been followed up at all in more recent years. This is an important line of research worthy of further exploration.

Virtual characters looking towards a participant is an important contributor towards the Plausibility Illusion (Psi) in VR^17^. This is the illusion that events depicted in the VR are actually happening even though the participants know for sure that this is not the case. Psi contributes to the likelihood of participants responding realistically to situations and events. For example Bergström et al.^46^ placed participants in an environment where a virtual string quartet was playing. It was found that Psi was greater in a condition where members of the string quartet would occasionally look towards the participant compared with a condition when this did not happen. Steed et al.^47^ placed participants in an environment with a group of virtual characters representing refugees waiting on a beach for a boat to take them from Turkey to Greece. In one condition some of the characters would return glances of the participant towards them, and in another condition would not. It was found that the gaze responsiveness enhanced Psi. Llobera et al.^31^ immersed participants in a virtual crowd walking towards a concert, and then amongst the concert audience. Again it was found that participants preferred a condition where the crowd members occasionally looked towards them compared with being ignored. In the bystander scenario two of the three requirements for Psi were satisfied: (i) since there was the conversation with V at the start of the scenario, where V spoke to the participant and maintained a dialogue, the virtual environment responded to the actions of the participant. (ii) Since the characters would occasionally look towards or talk to the participant there were events that spontaneously referred to the participant. Hence it is possible that our finding on the impact of the participant being looked at mostly by the victim, and secondly by the perpetrator, resulted in an increased level of Psi, which might be the reason why they were more likely to intervene in response to those actions, compared to a virtual bystander expressing his opinion. Of the 12 phrases used by the virtual bystander (Table 1) 10 were not addressed to anyone (e.g., “Someone needs to do something about this!”) and only 2 were directed at the participant (e.g., “Tell him to shut up!”). This may account for the order of probability distributions in Fig. 5.

It may be wondered why participants intervene at all in events that are not really happening, especially when once the argument between P and V started there was no response to their actions. We consider that in VR perceptions are first class meaning that a perception is a genuine perception irrespective of the source of the sensory data leading to the percept (whether it is reality or virtual reality)^48^. This corresponds to the view of the philosopher David Chalmers^49^ also argued for in his recent book^50^. That participants intervene in virtual environments has been observed for decades. For example, people become anxious in response to being near a virtual precipice^51^, they become anxious when giving a talk in front of a hostile virtual audience that does not respond to their actions^52^, they respond in the virtual Milgram Obedience scenario even though there is nothing that they can do can change the unfolding events except withdraw^11,12^. In a recent study, US police officers intervene to support a virtual victim of aggression by a virtual police officer even though their actions do not change the outcomes^53^. In another recent study people respond negatively to a hostile virtual crowd even though their own actions have no effect^54^. Moreover, in all our bystander studies cited in this paper participants intervene even though there is no response to their intervention. In VR people act on what they perceive, even though the source of the sensory data that leads to the perception is computer generated.

Although we used RL for this study, the problem is small enough (3 actions and 3 responses) that this was not the only viable method. A between groups factorial experiment could have been used with a single factor with 8 levels (1 with no actions, 3 with exactly one of the possible actions, 3 with exactly two of the possible actions, and 1 with all three actions) and then observing which condition resulted in the greater number of responses. Alternately each type of action could have been chosen randomly (as in one of the conditions of this experiment) and then a simple count of the numbers of responses to each. The advantage of RL is twofold. First, as we found in previous experiments, the number of participants needed for convergence seems to be less than the number needed for factorial studies—at least in this relatively simple set up. Though for a more complex setup with more actions and states, a factorial experiment would become prohibitive. The second reason is that the RL can be adaptive—perhaps different cohorts of people would respond differently to the actions. For example, here our sample consisted entirely of men, but women may respond differently. Or here our example consisted of Arsenal football fans, but people uninterested in football may respond to different cues. A vital question though is to know whether a greater number of actions (say, 10) would require a huge amount of data for convergence to take place. The small number of actions and responses is a limitation of our study.

Finally, we note that the method, especially if it is generalisable to a greater number of actions, could have implications for the behaviour of victims of aggression in a context where other non-involved bystanders are present. The conclusion of our proof-of-concept study would be that victims should attempt to involve bystanders by looking towards and explicitly calling for help. This removes ambiguity leaving onlookers in no doubt about what behaviour would be appropriate.

## Supplementary Information

Supplementary Information 1.

Supplementary Information 2.

Supplementary Information 3.
