# Supplementary material for: Encouraging bystander helping behaviour in a violent incident: a virtual reality study using reinforcement learning
Source: Sci Rep. 2022 Mar 9;12:3843. doi: 10.1038/s41598-022-07872-3 (PMC8907188; doi:10.1038/s41598-022-07872-3)
Supplement: Supplementary file 1 — Supplementary Information 1. [file 41598_2022_7872_MOESM1_ESM.docx]

# Supplementary Data

Supplementary Data S1 – This shows for each of the 45 participants the condition (Table 2), proprandom, and the number of responses out of 13 (resp).

Supplementary Data S2 – This shows each of the 13 actions over the 45 participants and the corresponding responses.
